# Open new possibilities in *Transplantation Research*

**DOI:** 10.1186/2047-1440-1-1

**Published:** 2012-04-24

**Authors:** Edward K Geissler, Alan Jardine

**Affiliations:** 1 Department of Surgery, University Hospital Regensburg, Regensburg, Germany; 2 Institute of Cardiovascular and Medical Sciences, University of Glasgow, Glasgow, UK

## 

The field of organ, tissue and cell transplantation through the recent decades has revolutionised treatment options for some of the most critically ill patients. Advancements in this discipline have been dramatic and provided life saving options, with a good quality of life, for patients with previously untreatable conditions. Both researchers and the public remain fascinated with the concept of transplantation because replacing body parts still seems to be part of science fiction, but yet is somehow possible through extraordinary pioneering research that has become part of routine modern medicine. Nonetheless, many serious issues remain unsolved limiting the further advancement of transplantation medicine, including the severe shortage of available organs/tissues and our inability to prevent immunological rejection without life-long suppression of general immunity with drugs that have substantial side-effects. Indeed, it could be said that the field of transplantation is presently resting uncomfortably on a plateau that most clinicians and researchers feel is far from where we would like to be [1]. Striving for adequate organ availability and preserving long term graft function are principal goals for the coming decades. This will require intensive and innovative basic and clinical research.

Providing new, reliable, rapid and fully open avenues for publishing transplantation research will only add to our ability to advance transplantation science and the care of transplant recipients. We believe that the advent of “open access publishing” brings a fresh perspective to publishing novel research; *Transplantation Research* aims to contribute to this *niche* by providing all the potential benefits that open access publishing has to offer. Among the advantages are speed of publication (published immediately upon acceptance), high visibility of your research (anyone with web access can read your article free of charge), and the provision of unlimited space for figures and extensive data sets (including video). With *Transplantation Research*, all articles will be rigorously peer reviewed by our outstanding global editorial board [2], and other *ad hoc* experts in the field. These combined values provided by open access publishing have been recognised by numerous societies [3,4], national authorities and institutions, many of which now cover publication costs that guarantee open public access [5]. For example, both of our own academic institutions are participants of BioMed Central open access publishing, covering all publication costs. In Germany, the primary scientific funding agency, the Deutsche Forschungsgemeinschaft (DFG), supports open access publishing by offering also to fund publication costs. Similarly, the European Commission strongly and actively advocates open access publishing of results from their funded projects. These are unmistakeable signals that the time and technology has come to bring our research results to all researchers, regardless of their local library funding resources, and to the general public. Moreover, it is recognition that public funding supports most research and that the public should have open access to the work they are paying for [6]. Therefore, we have taken on the task of establishing the journal *Transplantation Research* to help fulfil a mandate in transplantation to provide more full access to current knowledge in the field.

*Transplantation Research* is meant to be a portal for all aspects of transplantation investigation. We encourage the publication of studies involving a wide range of transplantation research, including: organ preservation, ischemia-reperfusion injury, histocompatibility, experimental immunosuppression, immune recognition - regulation, tolerance induction strategies, stem cell research for tissue regeneration and immune regulation, clinical trials, complications of transplantation (e.g. graft dysfunction, tissue repair, infections and posttransplant malignancy), biomarker investigation, organ donation and transplantation ethics, as well as xenotransplantation. We at *Transplantation Research* will encourage the publication of experimental research and pilot clinical trials that introduce new ideas and concepts to the field of transplantation. The introduction of novel experimental concepts that may have not yet been fully tested and early stage trial results are welcomed for publication and debate. In addition, we strongly encourage the submission of clinical trial protocols for publication; a publication portal for presentation and discussion of trial designs is largely missing in transplantation. Furthermore, we also encourage the publication of new and innovative experimental methods that can help other investigators establish reliable models and measures in their research. Finally, with the support of our publisher, BioMed Central, we will be adding a new publication media dimension to transplantation, by offering the possibility of a “video-type” publication (e.g. surgical methods, experimental procedures). Therefore, with the increasingly powerful platform of electronic open access publishing, we aim with *Transplantation Research* to offer all transplantation researchers new and innovative possibilities for publishing their basic and clinical studies.

There is also another perspective to consider – regarding the introduction of another journal in the field of transplantation. Simply stated, it is important that researchers have options to present their work to a different group of editors that inherently may consider studies to have more, or less, priority. How often have each of us sent for publication what we believed to be important and well-designed studies to one group of editors, only to be outwardly rejected, and turned around to send the work to another editorial group where the work was well received. It is not even uncommon for studies that were first sent for publication in a lower-ranking journal to be accepted later in a higher ranking journal, where the latter editors judge the study to be of a higher priority. This is normal and to be expected in the “business” of research publication; having multiple options, as long as journals use a solid and rigorous peer-review process, can only be positive for the development of the field.

We trust that our own expertise and views, and those of our editorial board, can indeed offer a fresh opinion and option for publishing your transplantation research. Moreover, we firmly believe that *Transplantation Research,* built on a foundation of an open access publishing instrument, also offers new publication categories, as well new innovative publishing formats, to transplant researchers. Importantly, we can ensure that the information is made freely available to all researchers, and the public sector. To us, this is a worthwhile endeavour that we hope will contribute to the improvement of outcomes following transplantation.
